# Meeting the challenges of the 21st century: the fundamental importance of trust for transformation

**DOI:** 10.1186/s13584-024-00611-1

**Published:** 2024-04-22

**Authors:** Martin McKee, May CI van Schalkwyk, Rachel Greenley

**Affiliations:** Department of Health Services Research & Policy, London School of Hygiene & Tropical Medicine, 15-17 Tavistock Place, London, WC1H 9SH UK

## Abstract

**Background:**

This paper is one of a collection on challenges facing health systems in the future. One obvious challenge is how to transform to meet changing health needs and take advantage of emerging treatment opportunities. However, we argue that effective transformations are only possible if there is trust in the health system.

**Main body:**

We focus on three of the many relationships that require trust in health systems, trust by patients and the public, by health workers, and by politicians. Unfortunately, we are seeing a concerning loss of trust in these relationships and, for too long, the importance of trust to health policymaking and health system functioning has been overlooked and under-valued. We contend that trust must be given the attention, time, and resources it warrants as an indispensable element of any health system and, in this paper, we review why trust is so important in health systems, how trust has been thought about by scholars from different disciplines, what we know about its place in health systems, and how we can give it greater prominence in research and policy.

**Conclusion:**

Trust is essential if health systems are to meet the challenges of the 21st century but it is too often overlooked or, in some cases, undermined.

## Background

This paper is one of a series examining challenges facing health systems in the post-COVID era. These can be thought of as a subset of wider challenges facing society. For example, the World Economic Forum (WEF) publishes an annual Global Risks Report [1]. In 2024, the greatest short-term (2 years) risks were, in order, misinformation and disinformation, extreme weather events, societal polarization, cyber insecurity, and interstate armed conflict. They echo a list of challenges identified previously by the Israeli writer Yuval Noah Harari [2]. He highlighted computer technology, disruption to financial, economic and political systems, polarized debates about key political topics such as immigration, our inability to come to terms with the end of many familiar stories about the world, forcing us to confront the reality of global complexities without clear narratives, our collective failure to give the next generation less information and more critical thinking abilities to assess the explosion of information, the erosion of privacy and individual freedom in the face of digital surveillance and data control by governments and corporations, and the era of “post-truth,” where objective facts are less influential in shaping public opinion than appeals to emotion and personal belief. Many of these were also brought together in an overarching framework of threats to health developed for the Pan-European Commission on Health and Sustainable Development [3]. 

We argue that trust, or lack thereof, in the people that we interact with at work and in other settings, in those in authority, such as politicians and official bodies, in those providing the services that we depend on, and in many other sets of relations underpins many of these issues.

In this paper, we focus on a narrower set of relationships that exist within health systems and, in particular, on the need for trust if we are to meet the challenges currently faced by health system and those that lie ahead. More than ever, health systems must continually transform to meet changing health needs and take advantage of emerging treatment opportunities. This has always happened; the rise of scientific medicine in the nineteenth century, creating a need for laboratories, imaging departments, and operating theatres transformed the hospital. However, the challenges we face today are characterised by increasing complexity. Team working is essential if we are to respond to this complexity, such as that associated with management of multimorbidity and frailty or the use of innovative therapies for pathological processes such as cancer. These require trust among all those involved [4]. It also encourages innovation, as individuals feel more secure when taking calculated risks [5]. 

The need for trust became particularly clear during the SARS-CoV2 pandemic, a time when health systems made remarkable transformations, repurposing facilities to expand intensive care capacity, using lay vaccinators in mass campaigns, conducting clinical trials at pace, and developing new ways to exchange knowledge. Yet they were making these transformations at a time when others were actively undermining trust, recalling several of the risks noted by the WEF, including disinformation, cyber attacks, societal polarization, and weaponisation of vaccines. Furthermore, not everyone benefited from such transformations.

Yet in the pandemic, trust (or lack thereof) had much wider implications [6]. Faced with images on television screens of hospitals being overwhelmed by those infected with a new and initially poorly understood virus [7], in many settings ordinary people turned to their political leaders to learn what they should do to protect themselves. Those political leaders turned to their scientific advisors, themselves often unsure about what to do. If those involved were to avoid uncertainty-induced paralysis they would have to trust one another. Mostly, this worked. Scientists worked at pace to assemble the best evidence they could, illustrating Harari’s point about the growing role of computers, while politicians listened to them, working out how to translate this evidence into policies that could be implemented. Together, they mostly, recognised that this would require measures that, even a few months previously, would have been inconceivable, such as lockdowns. Those who subscribed to the “post truth” ideas highlighted by Harari did not. Yet many politicians, perhaps reflecting on the trust imbued in them by the public, were surprised by how the public did trust health experts, for example reducing their movements before they were required to [8]. 

In this paper, we argue that trust is essential for the unprecedented transformations that took place at pace and scale during the pandemic, but also for the continuing, often incremental, transformations needed in the everyday work of health systems if they are to respond to changing population and patient needs and expectations. To transform in a way that people will accept and engage in, they must trust the motives and aims that guide such transformations, which must be undertaken in the interests of public health and equity. Trust is also essential in the relationships between health systems and the public they serve and the politicians who provide the resources they need to do so.

Before doing so, however, we should note that while this paper focuses on the trust that underpins interactions within the health system, this cannot be divorced entirely from trust more widely, especially in politicians and institutions [9] and in science [10] given their enmeshed relationship with health systems. During the pandemic, some politicians acted in ways that undermined the trust that was necessary for effective responses, saying and doing things that most people knew were wrong, whether it was advising use of bleach or ultraviolet light to kill the virus [11] or attending parties while instructing the public to adhere to lockdown and distancing regulations [12]. In one notable case, an advisor to the UK Prime Minister broke travel rules, which led to a significant drop in trust in the government. This decrease in trust was seen only in England and only towards the government, not the health services, suggesting the advisor’s actions directly caused the decline in trust [13]. Worse, there were many people who, for a variety of reasons, were actively undermining trust, whether because they genuinely believed in conspiracy theories, they wanted to undermine trust in democracy, or simply because they had found a way of making money from clickbait [14]. They benefited greatly from the increased reach of the internet, concerns about privacy, and adoption of “post truth” thinking, all as described by Harari [2]. 

Given these developments, health professionals have a duty to challenge those whose actions that undermine trust, such as populist politicians who show contempt for the truth [15], and to include explicit strategies to build trust and counter disinformation in health initiatives [16]. They are, in many cases, well placed to do so, with surveys showing that scientists and health workers are more trusted than politicians. For example, the Ipsos Global Trustworthiness Index finds doctors, on 59%, and scientists, on 58%, to be the most trusted groups, far ahead of politicians on 13% [17]. However some caveats are necessary. The Wellcome Trust Global Monitor, conducted by Gallup and the world’s largest study into how people around the world think and feel about science and major health challenges, shows considerable variation, with this trust substantially lower in many authoritarian countries where scientists may be seen as aligned with the regime [10]. Also, in some countries where public health measures have been weaponised, typically by populist right wing politicians, there is a large and growing partisan gap in trust in science [18]. This topic does, however, go beyond the scope of this paper.

## Main text

### Trust underpins relationships

Trust underpins all human relationships by influencing how the various parties interact. A trusting relationship is, inevitably, preferable to one characterised by distrust. In health systems, trust can be seen to underpin at least three important sets of relationships, while recognising the importance of other forms of relationships to the functioning of health systems [19]. 

First, there are signs, in some settings, of a loss of public trust that the health system will be there when they need it [20, 21]. These include people who are struggling to access care in overcrowded and crumbling health facilities, facing long waits and incurring substantial out-of-pocket payments. Their experiences leave them vulnerable to arguments by some that universal healthcare is somehow unaffordable or unsustainable, leaving some groups in the population, often those already disadvantaged, underserved.

Loss of trust is also manifested in attacks on health workers, such as those waged by individuals opposed to the measures that were necessary to interrupt transmission of SARS-CoV-2. These attacks have been facilitated by social media, spreading the disinformation that features in the WEF report [1] and Harari’s book [2] that seeks to undermine trust in science and in the health workers who use it to deliver evidence-based care.

Second, there are signs of a loss of trust among health workers in some settings. This is most common when they face low pay, unsupportive working conditions, lacking the tools they need to do their job. During the pandemic many experienced moral injury, historically associated with the inability to provide the care needed to those on the battlefield [22]. Many felt exhausted and demoralised and have seen too many colleagues become severely ill or die [23]. This feeling of neglect results in their departure from the health workforce, either to move abroad to where conditions are better or to seek other types of work. Others who stay may reassess their work-life balance, moving to part-time jobs. In the United Kingdom about one in three medical students does not plan to stay long term in the National Health Service [24]. This soon creates a vicious cycle as the work must still be done by a now depleted workforce. If they are to stay, they will need confidence that their working conditions can transform in ways that reflect their changing needs and allow them to deliver the quality of care they know should be possible.

Third, although difficult to measure, the failure of politicians in many countries to allocate sufficient funds for health care since the global financial crisis suggests that they may have lost trust in the ability of health systems to implement the transformations necessary to meet future challenges.

These problems are not new. They have been building up for many years, but a combination of developments means that they can no longer be ignored. The experience of the pandemic has shone a light on weaknesses in health systems and the limitations of current measures used to gauge health system functioning. As the population ages, fewer people are available to join the health workforce, while at the same time, there is a rise in the number of elderly individuals needing healthcare services. Geopolitical crises are fuelling inflation and disrupting supply chains, adding to the pressures on health systems.

### Trust in the clinical encounter and in society

The importance of trust can be seen not just at the system level but also in clinical encounter. Trust offers a feeling of security, fostering the connections that are vital in healthcare settings. Clear, honest, and transparent communication is easier when there is trust, important given that many of the problems that arise in health care are due to communication failures. Trust also promotes constructive dialogue, helping to resolve disagreements. Finally, trust fosters empathy, something that patients value highly [25]. 

Trust is indispensable if patients are to adhere to the medical advice and treatment necessary for their recovery. It encourages people to seek medical care promptly, preventing conditions from deteriorating and enhancing their experiences, a core aspect of system responsiveness, a primary objective of health systems. Trust is also a means to reduce uncertainty. When patients trust that they will get the treatment they need, they can have confidence in their future, providing an optimism that can thereby enhance wellbeing.

Health systems are embedded within societies, with further implications for health systems. Trust supports social cohesion, promoting cooperation and sharing of resources. Trust is also crucial for the well-performing economies necessary to generate the resources to pay for health systems. Environments characterised by high levels of trust enhance collaboration and teamwork. This is even more important with increasing engagement in the online world where people depend on strong and trusted institutions for protection from those who seek to exploit them.

In summary, the trust that the public places in healthcare professionals and the systems in which they work plays a crucial role in ensuring optimal health outcomes and positive experiences.

### Trust in data

Beyond the general issues outlined above, there are several specific areas where trust is important. One of the key lessons from the pandemic was the value of data from well-functioning information systems. In the United Kingdom, the OpenSafely system provided the platform for studies that provided rapid insights into what was working and, as importantly, what was not [26]. It is also intuitive that clinicians will make better decisions if they have comprehensive medical records, while surveillance activities, such as cancer registration, can be compromised where people opt out or where privacy restrictions make data linkage impossible. Yet, in some places, there has been a backlash against the collection of this information, in part reflecting a lack of trust that patient data will be adequately safeguarded [27]. This is not helped when the state takes actions that compromise individuals’ expectations of privacy, such the widespread use of facial recognition technology [28] or sharing of health data with immigration agencies [29]. However, there is a growing evidence base on what is needed to foster public trust in the collection of data by health systems and engaging with this research will be a critical element of health system transformation and trust building [30]. 

Another consideration is whether patients seek out clinicians or online resources as their first source of information, given the democratization of knowledge, brought about by the growth of the internet. This process has given birth to the “savvy” patient—a well-informed individual who critically evaluates health information before even setting foot in a physician’s office. Such a paradigm shift signals a departure from the era of implicit trust, where the professional’s education, training, and title were undisputed hallmarks of authority. The current landscape demands a recalibration of trust—one that is earned rather than assumed and recognizes the intricate interplay between empowerment and scepticism that modern patients rightly exhibit.

Similar concerns are arising in the relationship between staff and employers. Some employers in sectors such as retail distribution are now tracking movements of staff and thus their productivity. So far this is less common in health care but it is being introduced in some places to track those working in the community [31]. This risks eroding trust even further, especially when combined with algorithms that reduce health worker autonomy [32], exacerbating shortages in a sector that, very often, struggles to recruit and retain staff.

Finally, trust in information systems can also be damaged by hacking or cyberattacks. As with generation of disinformation, this can have many motives, from the use of ransomware to acts by hostile states [33], both high on the WEF list of risks [1]. 

The previous sections set out why one might expect trust to be important. But what does the evidence show?

### Research on trust

This section draws extensively on a recent systematic review by Taylor et al., which evaluates the body of trust-related literature within the healthcare domain over half a century [34]. The authors began by noting how the experience of the pandemic “has clarified the role that trust plays in virtually every element of healthcare delivery” [34]. Lack of trust delays care seeking and vaccine uptake. Healthcare professionals recognized the importance of being able to trust their colleagues, employers, and patients. Nevertheless, they also note how the literature on trust “can be as frustrating as it is voluminous”, with simple questions being met with complicated answers.

Taylor et al. categorize the literature into five distinct typologies: patient trust in clinicians, clinician trust in patients, intra-clinician trust, collective trust in healthcare organizations by patients and clinicians, and overarching trust in healthcare systems by patients, clinicians, and the broader populace [34]. The most substantial body of research pertains to the trust dynamic between patients and clinicians. A systematic review dating back nearly two decades underscores a dearth of empirical evidence substantiating the purported impact of trust on therapeutic outcomes and acknowledges limitations of subsequent research, primarily dominated by cross-sectional surveys, qualitative interviews, and a handful of interventional studies [35]. 

The situation has since improved. A more recent systematic review, although still a decade old, that focused on generalized trust, as opposed to clinician-specific trust, included thirteen randomized controlled trials evaluating ways to foster trust [36]. They included strategies such as enhanced communication, motivational interviewing, shared decision-making, patient-centred care, empathic care, and cultural competency training. Collectively, these interventions demonstrated a modest yet statistically significant improvement in healthcare outcomes, including alleviation of pain and anxiety and improved diabetes management.

Some literature explores the less frequently examined perspective of clinician trust in patients, highlighting a reciprocal vulnerability, where the professional reputations of clinicians are threatened, for example by malicious online reviews [37]. This underscores the necessity for innovative methodologies that can elucidate the evolution of trust in protracted clinician-patient relationships, particularly for chronic conditions.

There is also a scant literature on clinician trust in their employing institutions, some of which invokes the concept of institutional betrayal, where organisations or authorities fail to protect their staff [33]. This became an issue during the pandemic when health workers were struggling to obtain personal protective equipment, especially when they became aware that well-connected individuals were profiteering from the shortages [38]. 

Studies on trust in health systems are divided into those looking at trust in specific systems and more generalized health system distrust [39]. While there is a substantial volume of literature assessing trust in particular systems, primarily through public opinion surveys, exploration of the causative factors behind system distrust is less developed and predominantly from the United States, limiting its global applicability.

In summary, while the body of research investigating trust within healthcare settings is burgeoning, significant gaps remain. These gaps necessitate further scholarly efforts to elucidate the intricate linkage between trust and health outcomes and, crucially, strategies to cultivate and sustain trust among all groups within the healthcare milieu. The next section moves to the second T, transformation.

### Transformation

The burden of disease is changing, with the growth of multimorbidity and the emergence of new diseases, such as COVID. The opportunities to intervene are changing. The 2023 Nobel Prize in Medicine, awarded to Katalin Karikó and Drew Weissman, recognised work that contributed to the RNA vaccines [40]. This has the potential to be a game-changer for some cancers. Public expectations are also changing. The traditional paternalistic relationship between the doctor and the patient has mostly gone in many contexts, arguably not soon enough.

Trust is essential if the complex transformation of health systems needed to keep up with these changes is to be achieved. This does, however, raise certain questions: What type of health system transformation is necessary, and how can it be actualized? Which components of the health system are fostering trust, among whom, and which are eroding it? Who holds the keys to strengthening public trust, and does this differ across diverse communities? These are questions that cannot be answered in detail here and will differ from health system to health system. If trust is the problem, then what is needed is a radically new approach, placing the patient, in some cases accompanied by her carer, at the centre. But this does not mean that the patient should be left to sort themselves out. Rather, the models of care that emerge must come from a sharing of knowledge and experience, and mutual respects for each other’s expertise, something that will not be easy.

The challenge is to get the right mix of health workers, with the right skills and technology, including medicines, in the right facilities, in the right place, at the right time to meet the needs of every patient. If this is to happen, those in charge of the health system must be incentivised, encouraged, and supported to work with patients, carers, families, and communities to co-create solutions, while those at higher levels of the system must facilitate this process [41]. This requires a completely new approach to health systems, based on a commitment to meaningfully include all stakeholders, invest the resources needed for change, and innovate with new models of care.

So how can this be brought about? The first step is to challenge the sense of pessimism that has afflicted many health systems. There are many examples of innovative solutions that can be learnt from. Some of the most imaginative address the needs of those living in places with low population densities or groups who have fallen through the cracks in existing systems.

It is also necessary to think again about what health professionals do. Task shifting has been discussed for decades but, for most of that time, it has been viewed too simplistically. Too often it has been portrayed as a means of cost-cutting, getting less skilled people to take on roles undertaken by health professionals. It is much more complicated and should be guided by an ambition to improve patient care and trust. Task shifting involves not only moving tasks between different types of health worker but also between health workers and patients and their carers and, increasingly, between both groups and machines [42]. 

Crucially, these solutions are not only needed for complex problems. Managing hypertension should be easy. With automatic sphygmomanometers, anyone can measure blood pressure and there have been safe and effective drugs for 60 years. So why is it so difficult? Some recent studies asked people in some of the poorest communities in middle-income countries about the barriers they face [43, 44]. They spoke of how what should be a simple series of clinical encounters can be extremely complex, a situation not helped by patients and health workers having quite different understandings of the issues. In the HOPE-4 trial, researchers spent a year working with marginalised communities in Malaysia and Colombia to develop an intervention tailored to their circumstances [45]. The details differed in each country but both involved mid-level health workers, simplified guidance on tablets, combination therapy, and peer support. The results were much better than anticipated. This was because of the time spent working with the people concerned. However, these principles can be applied to more complex problems, such as the engagement of people with lived experience of psychosis in sub-Saharan African countries in the SUCCEED project [46]. 

Another contemporary example is research on cervical cancer prevention, where results in many countries are terrible. HPV testing, based on self-sampling, offers a possible response for disadvantaged women who are not reached by screening programmes. An ongoing study is working with such women in several countries in Europe to find solutions that meet their needs [47]. This can be as simple as finding a way that they can return samples when the postal system does not work.

A word of caution is, however, needed. The UK is experimenting with a new model of task shifting. Faced with a drastic shortage of doctors, it has introduced a new type of worker, the physician associate. Superficially, the idea may seem appealing. Doctors spend much time on tasks that do not require their skills, whether administrative or clinical. Physician associates complete a two-year training course. Some have previous experience as other types of health worker, such as paramedics, but this is not a mandatory requirement. For now, they are not allowed to prescribe or order tests requiring exposure to ionising radiation. But that is about the extent of the restrictions placed on their practices. Some are reported to be performing surgery and others seeing unselected patients in general practice [48]. In theory they are supervised by a doctor but it is unclear if this is happening in practice, especially when they are on the same rota as doctors [49]. Some employers are describing them in ways that seem designed to create confusion for patients who do not realise that they are not seeing an actual doctor, something that is likely to place public trust at risk. Many are paid much higher salaries than the doctors they are meant to be assisting, something that seems counterintuitive until it is realised that they cost the hospital less than employing locum doctors from an expensive employment agency. However, the salary differential, coupled with the loss of training opportunities for doctors, illustrates why it is so important to consider unintended consequences of what seem like intuitively good ideas.

Gilles, who argues that trust is at the core of successful reform of health systems, contends that leaders must be facilitators, supporting both caregivers and care recipients to craft an environment where trust can flourish [30]. In doing so, they must ensure that trust is not a static credential, but a dynamic asset continually cultivated through transparency, reliability, and responsiveness to the changing needs of society. Thus, trust is not merely a component of transformation; it is the foundation upon which successful and sustainable transformation must be built and maintained.

There is one other area arising from the growth of computing power discussed by Harari [2] that requires particular attention. Artificial intelligence systems can now perform close to, or in some cases as well as, trained clinicians. They do best in areas that depend on pattern recognition, when looking at images in radiology, histopathology, or dermatology. Some research has found that patients even prefer responses to medical questions from a chatbot to those from a physician, but clearly context matters [50]. Yet, once again, caution is needed [51]. Algorithms developed using data from one population may produce misleading results when applied to another [41]. Algorithms may also reproduce existing biases in treatment when, for example, they use subtle clues to determine a patient’s race in a setting where there are already racial biases in treatment decisions [52]. One study from the United States found that a white patient with a given profile was given a lower risk than an otherwise similar black patient because the algorithm considered the cost of treatment [53]. The algorithm was just reflecting the reality. Black patients receive less expensive care. A further concern is that dependence on Artificial Intelligence may lead to deskilling and loss of experience among the current generation of trained clinicians [54]. It will then be difficult to identify when algorithms do go wrong. Finally, there is inherent uncertainty in health care but when two clinicians disagree, they can often resolve the issue by discussion. At least for now, one cannot have such a conversation with a machine [55]. 

Trust also influences interactions between health professionals and artificial intelligence algorithms. If they do not trust the algorithms, they will not use them but if they trust them too much, referred to as misplaced trust, they might ignore their own better judgment because of what the algorithm produces. This problem is hard to solve because the way that machine learning works is often a mystery. They are like “black boxes” in that it is not possible to see inside and understand why they make certain decisions. This happens for three main reasons: companies want to keep their methods secret, users do not always understand the technology, and the programmes themselves are very complex [56]. An example of how things can go wrong is a study where an artificial intelligence system was supposed to recognize pictures of horses. It did not; instead, it identified horses by the presence of a small copyright tag in the pictures used [57]. When this tag was put on other things, like cars, the computer mistakenly said they were horses too. The implications for this type of error within health care delivery could be devastating.

### Going forward

This paper has argued that trust is important, even if it is often overlooked in discussions about health system reform. If it is to be taken seriously, it must be measured, with the results used to determine the success or failure of those reforms and associated transformations of the delivery of care. This speaks to issues of computing power but also privacy. This must include all three types of trust discussed previously. These measures must also be included in the routine monitoring of health system performance accompanied by greater efforts to understand the mechanism underpinning processes that build or erode trust.

But how? Like many things that are important, such as love or friendship, one knows when it is there and when it is not, but it can be difficult to measure and maintain. Similar issues arise with trust. Even though the term trust is widely used, definitions of trust and trustworthiness, are contested, with major differences among disciplines, which often remain siloed. Consequently, there are major methodological challenges [58]. 

Second, trustworthiness cannot be fully observed by either those whose trust is sought or by the researchers seeking to understand it. While a patient may have a well-founded expectation that a clinician will treat them well, they cannot predict the future so their perception will be influenced by their attitude to risk. It cannot be assumed that two people recording scores of four on a five-point scale of trust mean the same thing.

This does not mean that one should give up. Much can be done with relatively simple instruments. Political scientists have long been asking people about their confidence and trust in institutions, including health systems [59, 60]. It does get more difficult when one wants to understand the factors influencing trust and how it manifests. Fortunately, there are several validated scales [39]. They typically include concepts of honesty, communication, confidence and competence, although fidelity, system trust, confidentiality and fairness also feature, even if less often. The three that are now most widely used are the Group-Based Medical Mistrust Scale, Medical Mistrust Index, and Health Care System Distrust Scale [61]. However there is still much to do to understand fully their psychometric properties, especially in different cultures, and to improve them. The Peoples Voice Survey is a novel survey being conducted across 15 countries and is specifically designed to measure people’s confidence in their health systems, an important component of trust [62]. To be able to trust their health system people need to be confident that it will provide them with health security, meaning that they believe the health system can provide them with the care they need, that they can afford that care and that they will receive quality care. This type of innovative approach holds great promise for the study of people’s trust in their health systems ability to provide them with quality and affordable care.

## Conclusion

Health systems, like society in general, face many challenges. Several of the greatest challenges have important consequences for trust, a phenomenon that, as we have argued, is fundamental to the operation of health systems and their ability to transform in response to changing circumstances. This includes the trust of the public, the patient, the health worker, and the politician. All are as important as the other and health systems much be transformed in order to ensure they are both trusted and trustworthy. If the trust that is so important for these relationships can be restored, health systems will be in a much better place. Patients will value services that are designed to meet their needs and expectations rather than those of the healthcare providers. The health workforce, who have chosen their careers because they want to provide appropriate and compassionate care, will feel supported and valued and be more likely to stay. Politicians will feel that the investments that they might make in health systems will be used in ways that make a real difference to their public.

But it is also important to remember the second T, transformation. This will only work if it is based on the concept of co-creation through genuine and meaningful engagement with people and communities. This means that the problems should be identified and articulated by those on the frontline, both patients, the wider public and professionals, with the role of health authorities and governments being to support the development and implementation of solutions, recognising the inevitable resource constraints. In this way, those involved will have confidence in the innovation and trust in its implementation.

## Data Availability

Not applicable.
